# Head-to-head Comparison of Transrectal Ultrasound-guided Prostate Biopsy Versus Multiparametric Prostate Resonance Imaging with Subsequent Magnetic Resonance-guided Biopsy in Biopsy-naïve Men with Elevated Prostate-specific Antigen: A Large Prospective Multicenter Clinical Study

**DOI:** 10.1590/S1677-5538.IBJU.2020.06.08

**Published:** 2020-09-02

**Authors:** Felipe Lott

**Affiliations:** 1 Instituto Nacional do Câncer Rio de JaneiroRJ Brasil Instituto Nacional do Câncer – INCA, Rio de Janeiro, RJ, Brasil

Marloes van der Leest ^1^, Erik Cornel ^2^, Bas Israël ^1^, Rianne Hendriks ^3^, Anwar R Padhani ^4^, Martijn Hoogenboom ^1^, et al.

^1^ Department of Radiology and Nuclear Medicine, Radboud University Medical Center, Nijmegen, The Netherlands; ^2^ Department of Urology, Ziekenhuis Groep Twente, Almelo-Hengelo, The Netherlands; ^3^ Department of Urology, Radboud University Medical Center, Nijmegen, The Netherlands; 4 Paul Strickland Scanner Centre, Mount Vernon Cancer Centre, Northwood, UK

Eur Urol . 2019 Apr;75(4):570-578.

**DOI: 10.1016/j.eururo.2018.11.023. | ACCESS: 10.1016/j.eururo.2018.11.023**

## COMMENT

The use of multiparametric magnetic resonance imaging (mpMRI) reduces the insignificant and increases the significant prostate cancer detection (1).

This is level 1a evidence study included patients between 50-75 yr with a PSA > 3ng/ml. All men underwent mpMRI.

The results shows that the mpMRI pathway is noninferior to the ultrasound guided biopsy pathway in discover significant prostate cancer (sPCa) and is superior for detecting fewer insignificant cancers. So, it supports a no immediate biopsy approach for men with a non-suspicious mpMRI, missing only 4% of sPCa and avoiding 2.9% of complicated sepsis related to biopsy. This trial showed 2 times avoidance biopsy rates if compared with the PROMIS and the PRECISION trials (2, 3).

